# Using UAV Photogrammetry to Analyse Changes in the Coastal Zone Based on the Sopot Tombolo (Salient) Measurement Project

**DOI:** 10.3390/s20144000

**Published:** 2020-07-18

**Authors:** Pawel Burdziakowski, Cezary Specht, Pawel S. Dabrowski, Mariusz Specht, Oktawia Lewicka, Artur Makar

**Affiliations:** 1 Department of Geodesy, Faculty of Civil and Environmental Engineering, Gdansk University of Technology, Narutowicza 11–12, 80-233 Gdansk, Poland; 2 Department of Geodesy and Oceanography, Gdynia Maritime University, 81-347 Gdynia, Poland; c.specht@wn.umg.edu.pl (C.S.); p.dabrowski@wn.umg.edu.pl (P.S.D.); o.lewicka@wn.umg.edu.pl (O.L.); 3 Department of Transport and Logistics, Gdynia Maritime University, 81-225 Gdynia, Poland; m.specht@wn.umg.edu.pl; 4 Department of Navigation and Hydrography, Polish Naval Academy, Smidowicza 69, 81-127 Gdynia, Poland; a.makar@amw.gdynia.pl

**Keywords:** photogrammetry, UAV, coastal line, tombolo, salient, Sopot

## Abstract

The main factors influencing the shape of the beach, shoreline and seabed include undulation, wind and coastal currents. These phenomena cause continuous and multidimensional changes in the shape of the seabed and the Earth’s surface, and when they occur in an area of intense human activity, they should be constantly monitored. In 2018 and 2019, several measurement campaigns took place in the littoral zone in Sopot, related to the intensive uplift of the seabed and beach caused by the tombolo phenomenon. In this research, a unique combination of bathymetric data obtained from an unmanned surface vessel, photogrammetric data obtained from unmanned aerial vehicles and ground laser scanning were used, along with geodetic data from precision measurements with receivers of global satellite navigation systems. This paper comprehensively presents photogrammetric measurements made from unmanned aerial vehicles during these campaigns. It describes in detail the problems in reconstruction within the water areas, analyses the accuracy of various photogrammetric measurement techniques, proposes a statistical method of data filtration and presents the changes that occurred within the studies area. The work ends with an interpretation of the causes of changes in the land part of the littoral zone and a summary of the obtained results.

## 1. Introduction

Unmanned platforms (water, land and air) are devices capable of moving in a specific environment without the presence of an operator on board [1,2]. Their movement can be remotely controlled by a human being or programmed and executed automatically. Their main advantage is their ability to perform their task in areas where a manned mission would be difficult or impossible. Researchers and engineers soon noted these properties and started to use unmanned vehicles as mobile platforms for research equipment [3,4,5,6,7,8,9]. As a result, it enabled research in new locations and with unprecedented frequency.

The coastal zone is a transition zone between the land and the water environment [10]. The analysis of changes taking place in this zone requires the application of various measurement methods and techniques capable of drafting three-dimensional environmental models [11,12]. Techniques of terrestrial, airborne or mobile laser scanning and satellite, aerial or low ceiling photogrammetry are currently used to record the shape of the land surface [6,7,10,12,13,14,15,16,17,18,19,20,21,22,23,24]. Low-cell photogrammetry has become a very good source of morphological data in the coastal zone. The paper [25] compares the differences between the anthropogenic and the natural coastal zone, based on the morphological reconstruction of dunes, using a low-cost unmanned aerial vehicle (UAV) and the photogrammetric reconstruction method, in which the authors were able to provide high resolution digital surface model (DSM). In the study [26], the researchers examined the applicability of UAVs and structure from motion (SfM) algorithms to reconstruct the costal environment. Authors compared the models from terrestrial laser scanning (TLS) and UAVs, and then generated high resolution DSM form the combined data. Single or multibeam echo-sounders are used for recording the shape of the bottom surface of a body of water [27,28,29,30]. Multibeam echo-sounders (MBES), which were originally designed for deep-water measurements, are commonly used to obtain high-resolution bathymetric data in coastal areas [28,30,31]. Recording the shape of both surfaces at specific time intervals and comparing the generated spatial models allows one to analyse the time changes that occurred in the environment and determining their dynamic characteristics [32,33].

The main factors influencing the shape of the beach, shoreline and seabed include undulation, wind and coastal currents [34,35]. Beaches are constantly transformed by waves and wind, and the material forming them is subject to constant movement from the sea to land and back [10,36]. Incoming waves cause an ascending movement, whereas return flow causes a descending movement. If the waves hit the shore at a certain angle, then the movement to and from the shore overlaps with the movement of grains along the coast. In addition, in the moderate climate zone, winter is characterized by frequent storms. Therefore, shore erosion dominates during this period, whereas in summer, shore deposition prevails. Additionally, these changes heavily depend on human activity in the coastal zone involving consisting of the construction of infrastructure and other facilities [19,37]. Coastal currents in this area that displace bottom sediments are responsible for creating links between the mainland and coastal islands or hydro-technical structures. These forms are called salient or tombolo [38,39,40,41,42,43]. Tombolo or salient is an accumulative form of coastal relief closely related to the influence of coastal currents. The littoral transportation is decreased due to the attenuated wave and longshore currents in the area sheltered by the breakwater. The material is deposited and it gradually reaches more and more towards the island or structure (pier or breakwater). Depending on the conditions, the trapping sand will develop into a tombolo or salient. If the length of the pier is equal to or longer than 0.8 times the distance between the shore and the breakwater, tombolo will be formed. Here, in the case of the Sopot pier, the breakwater length and its distance do the shoreline ratio is equal to 0.78, hence it is likely that a tombolo will eventually develop. Furthermore, the desire to form a tombolo in this area is constantly stopped by active human activity through regular removal of accumulated deposits by machines (excavators, etc.). At this stage, the presented form is the salient; however, parameters other than the breakwater length and distance may influence the accumulation pattern. Apart from that, these phenomena cause continuous and multidimensional changes in the shape of the seabed and the Earth’s surface, and when they occur in an area of intense human activity, they should be constantly monitored [44]. Tombolo or salient is a local phenomenon that allows for use of unmanned platforms with measuring equipment for testing.

**Figure 1:**
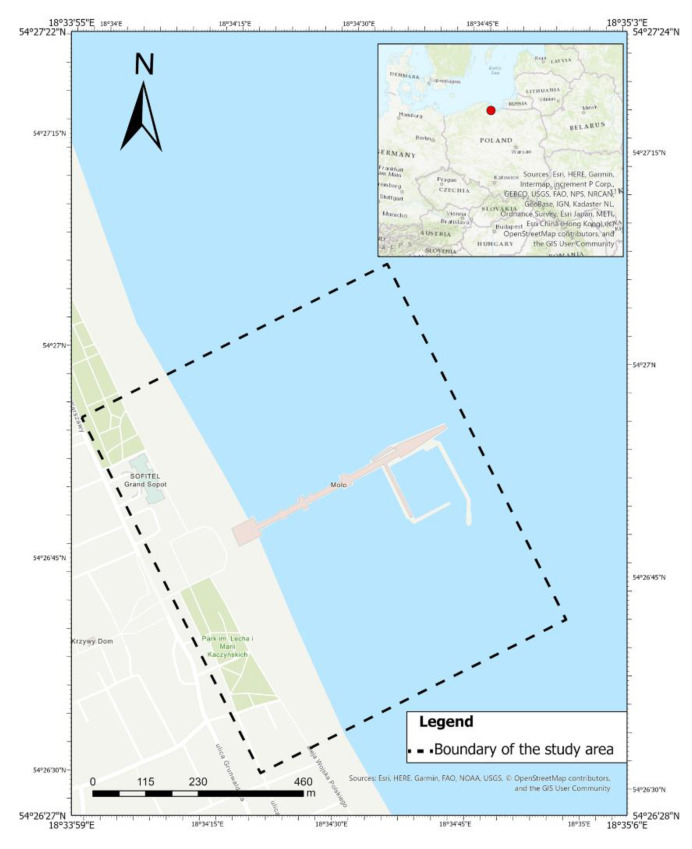
Study area-location: city Sopot, Poland.

Sopot, a city located in northern Poland on the Gdansk Bay, is a popular tourist resort (Figure 1). In the coastal zone of the city, there is a platform perpendicular to the shoreline, adapted to serve sports vessels and small passenger ships. In this area, tombolo, salient and changes in land structure influence human activity. A shoal patch in the area, due to sediment transport, poses a threat to the traffic of ships and tourist vessels. The movement of sand on the beach makes it necessary to keep it in a condition suitable for tourists. Periodic analyses and identification of processes that take place in this area allow for accurate planning of anthropopressure [44]. For this purpose, we developed and implemented a complementary methodology of evaluation of the phenomena occurring within the littoral area using unmanned platforms [42].

The research [42] presents the genesis of tombolo (salient) formation in Sopot in great detail and describes the methodology of integrated spatial measurements of this phenomenon, and finally, it proves the accuracy of measurement techniques used. This study [42] uses a unique combination of bathymetric data obtained from a hydrographic motorboat to the 0.6 m isobath, unmanned surface vessel (USV) to the 0.2 m isobath, photogrammetric data obtained from unmanned aerial vehicles (UAVs) and terrestrial laser scanning (TLS), and geodetic data from precision measurements with receivers of global navigation satellite systems (GNSS). This great variety of different sources of spatial information allows one to gain very detailed knowledge about changes taking place in the environment. On the other hand, it entails several issues related to the interoperability of spatial data sets and their harmonisation.

This paper focuses on photogrammetric measurements from unmanned aerial vehicles for the measurement campaign of the unique tombolo (salient) phenomenon in the studied area (Figure 1). The article discusses the procedure of developing photogrammetric data from two different measurements carried out by unmanned aerial vehicles. Measurements using UAV photogrammetry were aimed at measuring morphological changes occurring within the beach. Within the beach, two oppositely motivated pressures are stumbling. The environmental forces that naturally form the surface of the beach and the human pressure that continuously adjust the area to the requirements of the touristic activity. In order to reveal changes within the beach surface, the prepared point clouds were initially filtered with statistical methods. This operation minimized outlying points and allowed to precisely align the point clouds. As a consequence, the changes were calculated using distances directly between two point clouds. Detailed elements of the procedure have been described and presented in the article. The publication has been divided into six sections. The first section is the Introduction, which presents the motivation for this study. The second section, Materials and Methods, describes the tools and methods used to process the data. The third section discusses the results obtained. The paper ends with a Conclusions section which summarizes the most important aspects of the study.

## 2. Materials and Methods

Tombolo (salient) measurement campaigns in Sopot spanned three years. The most important measurements realised with a full range of measurement methods and the use of unmanned systems, took place in November 2018 and November 2019 [42]. The article covers this two-year period, where measurements were made with a full range of unmanned methods and in accordance with the developed methodology presented in [42]. The dynamic development of UAV technology during this period resulted in the use of various unmanned platforms equipped with various cameras.

### 2.1. Data Acquisition Process

A photogrammetric flight was performed in 2018 with a type DJI Mavic Pro UAV, while in 2019, a DJI Mavic Pro 2 was used for image acquisition. Aircraft of this type are commercial flying platforms designed and intended mainly for recreational flights and amateur filmmakers. The photogrammetric community soon appreciated the versatility and reliability of these devices. They gained popularity mainly due to their simplicity and intuitive software. The technical data of both platforms are presented in Table 1.

Unmanned aerial vehicles (UAVs) are equipped with stabilized visible light cameras of type F230 and L1D-20C with 12 and 20 million pixels, respectively. The technical data of both cameras are presented in Table 2.

In terms of photogrammetry, the coastal zone combines a land rich in solid textures with water that is extremely variable in terms of images and very luminous. In this case, the UAV measurement focused on the beach, which was considered a priority area, with the water area and the offshore pier being treated as auxiliary areas. It was assumed that a ground sampling distance (GSD) of approximately 2 cm/pixel would be sufficient to generate a numerical model of the terrain and point clouds of the land surface, and it would allow for further analysis of the phenomena with satisfactory accuracy. Based on this value, the height of the flight over the beach was determined. No required minimum GSD values for the auxiliary area were assumed.

The data of the planned flight patterns are presented in detail in Table 3. Because the parameters of the cameras used in both campaigns differ (Table 2) for a fixed GSD, the average flight altitude ($h_{MAGL})$ over the priority area was calculated using the formula:

(1) $$h_{MAGL}=\frac{I_{W}\;GSD\;F_{R}}{100\;S_{W}}$$

where, $I_{W}$—image width expressed in pixels (px), GSD—the given ground sampling distance in pixels per centimetre (px/cm), $F_{R}$—actual focal length of the camera (mm) and $S_{W}$—actual sensor width (mm).

**Figure 2:**
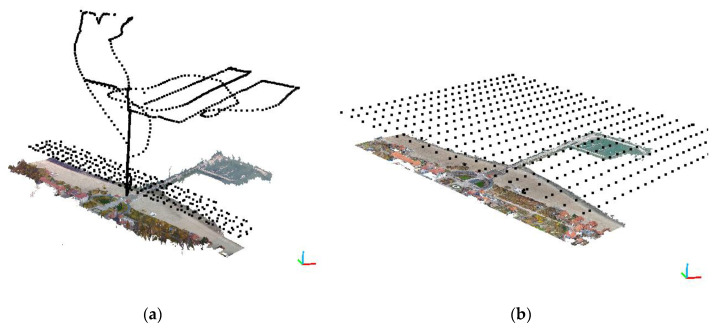
Location of cameras (black spots) and the implemented flight plan (**a**) flight 2018 (**b**) flight 2019.

Flights performed in the 2018 campaign consisted of two different plans. The first plan, based on the double grid scheme [45], was realized in automatic mode over the priority area. This scheme is mainly used for modelling urban areas, where it is important to obtain information on the faces of buildings or areas with very variable relief. The land area was covered twice with a demanding and relatively long flight. Such a way of taking pictures minimizes information loss, but the flight consumes more energy of the main UAV battery and takes longer. The second scheme was implemented in manual mode. In this case, the operator manually controlled the UAV over the pier structure and over the sea area (Figure 2a). As it was assumed, the additional area, relevant for the model as a whole, did not require modelling with a pre-determined minimum ground sampling distance (GSD). The absence of this limitation allowed for a significant increase in flight altitude and, as a consequence, obtaining a larger sea surface image visible on the orthophotomap.

The photogrammetric flight in the 2019 campaign was planned and executed according to the single grid plan [45]. Due to the size of the area and the wind blowing at the speed of over 5 m/s, the planned flight time exceeded the maximum safe time for the unmanned aircraft used. Therefore, it was decided to divide the research area into three smaller ranges. This enabled making three safe flights lasting 18 min and to completely cover the area under study. In this case, the measurement was performed at a fixed altitude, which did not allow for depicting a sea area as large as in the first case.

When analysing the results, note that no tie points can be found on luminous and non-textured surfaces [46]. Examples of such surfaces include water, snow, glass walls of high-rise buildings or windows. This makes it impossible in practice to generate a point cloud or orthophoto of these surfaces [47,48,49,50]. In such cases, an increase in flight altitude is applied which, in turn, allows to extend the terrain size of the photo and illustrate a larger terrain context. Then, at the expense of loss in visible texture details and geometric quality of the model, it is possible to develop a photogrammetric product [51]. Such recommendations are included in the Pix4D software documentation [52]. This method was used for the 2018 campaign. The auxiliary area was covered with photos taken at altitudes up to 260 m.

### 2.2. Processing of Photogrammetric Data 

The result-processing stage starts with importing all of the photos to computer software and entering the processing settings. Different commercial photogrammetric software was used for both cases, Pix4D Mapper and Agisoft Metashape, respectively. The project calculation for both cases was done differently with varied user access to the initial processing settings. Each manufacturer also uses its own, different file formats, which are not mutually compatible. This implies the necessity to make data processing and exchange uniform.

**Figure 3:**
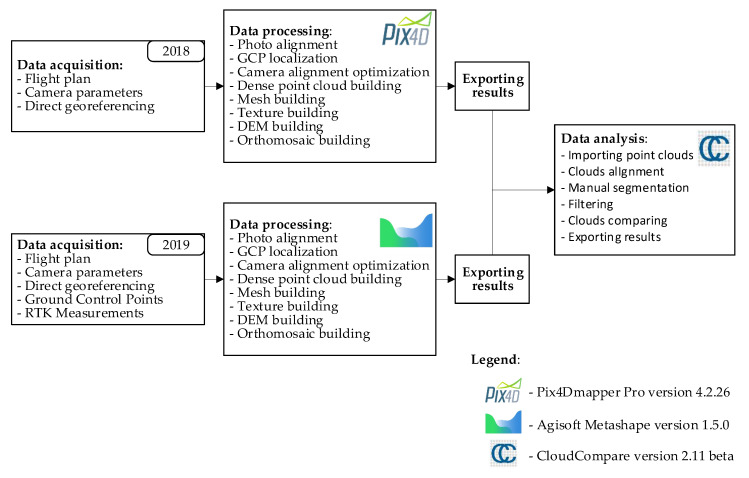
Data processing.

Figure 3 presents the photogrammetric process used to study the presented phenomenon. An analogue algorithm was used in the studies [53], which modelled the topography of a quarry with the following initial rules assumed:

1. application of georeferencing directly and/or through the use of ground control points, standard processing settings (as proposed by the software);
2. exporting results in the form of a high-density point cloud to LAS format, a surface model to OBJ format and a numerical coverage model and orthophotomap to TIFF format;
3. the UAV navigation system records camera position relative to the WGS84 ellipsoid and represents it with geodetic coordinates *B*, *L* and *h* (latitude, longitude, ellipsoidal height);
4. position of the photogrammetric warp points is expressed in coordinates in the Polish PL-2000 system of flat coordinates, and their altitude is expressed relative to the quasigeoid in the Polish PL-EVRF2007-NH altitude system;
5. the location of ground control points is measured with an accurate method of differential satellite positioning GNSS RTK (accuracy 2 cm, *p* = 0.95);
6. the Polish PL-2000 flat coordinate system is the target coordinate system of the study;
7. altitudes are related to the quasigeoid PL-EVRF2007-NH.

**Figure 4:**
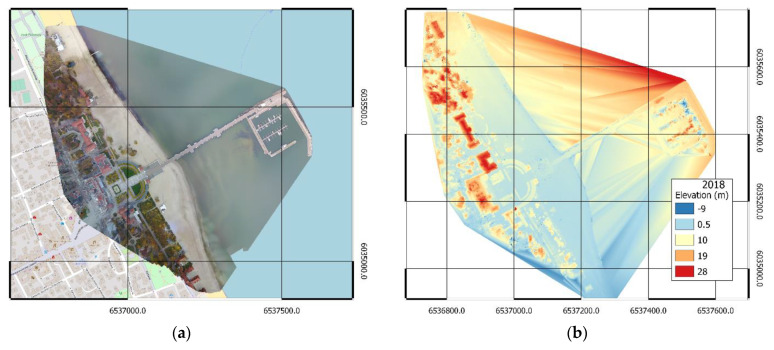
Orthophotomap (**a**) and DSM (**b**) of the area adjacent to the pier in Sopot, measurement of 2018.

**Figure 5:**
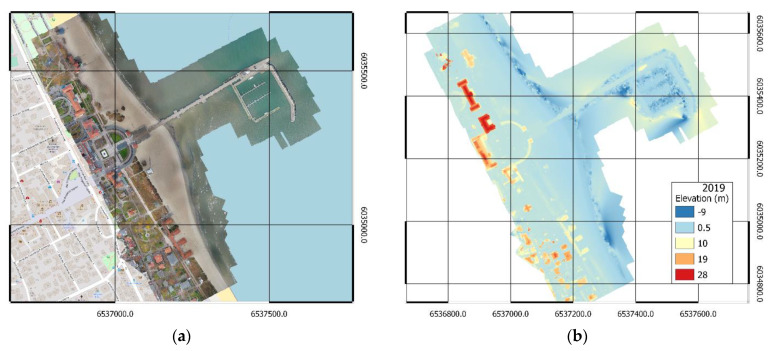
Orthophotomap (**a**) and DSM (**b**) of the area adjacent to the pier in Sopot, measurement of 2019.

The results of handling data from photogrammetric measurements of the 2018 and 2019 campaigns in the form of orthophotomaps are presented in Figure 4a and Figure 5a, respectively. Numerical land cover models are presented in Figure 4b and Figure 5b, respectively. In addition, high-density point clouds were generated for further comparative analyses. 

### 2.3. Accuracy Characteristics of Photogrammetric Studies

Details on measurement conditions in 2018 and 2019 have been compared in Table 4. Please note that the measurement accuracy reports generated by Pix4D and Agisoft Methashape software differ in terms of content and data presentation. For this study, the values given in Table 4 have been recalculated and presented in uniform units to enable comparative analysis.

In both measurement campaigns, the UAVs used could record images with metadata on the current UAV position. These data are saved in EXIF (exchangeable image file format). The current position, recorded by an onboard GPS receiver and saved in the image metadata, can be compared with the external orientation elements (EOP) determined at the aero-triangulation stage. In this way, for each image, the absolute position error of the central projection position was determined in meters and the standard deviation of the position error for the entire block of images was calculated. The 2018 data presented in Table 4 indicate low error values in the horizontal plane (*x*, *y*) and vertical plane (*z*) and low standard deviation (σ), which proves that position measurements are very stable. On this basis, it is concluded that georeference for each image was determined correctly, with high accuracy and there were no significant deviations. The values were within the accuracy range typical for single-frequency GPS receivers [54,55,56,57,58]. 

**Figure 6:**
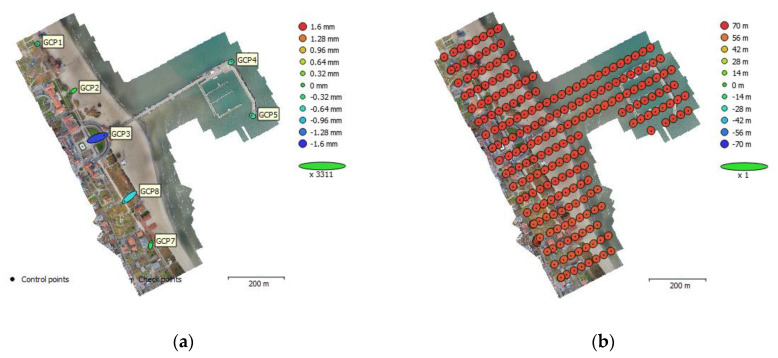
Ellipses of GCP (**a**), and EOP (**b**) location errors.

External orientation elements specified for the 2019 flight have a larger mean absolute camera position error. These values result from the use of ground control points in the photogrammetric process and determining their position in relation to another reference system. The measurement performed by an on-board GPS records the position and altitude relative to the WGS84 ellipsoid. The GCP position was determined relative to the Polish PL-2000 flat coordinate system and the altitude relative to PL-EVRF2007-NH quasigeoid. As presented in Table 4, the error values in the horizontal plane (*x*, *y*) are within the range typical for GPS receivers used in commercial UAVs. The absolute error in the vertical plane (*z*) is significantly greater (65.4 m) and results directly from using different altitude reference systems. The determined ellipse errors for EOP are shown in Figure 6b. For DJI Mavic Pro 2, a significantly smaller standard deviation of the recorded photo position was observed.

The photogrammetric warp points were distributed evenly over the entire area of the study and their position was measured with an accurate GNSS RTK satellite positioning method. Five points were located on stable infrastructure elements, such as concrete sidewalks running along the beach. Two more were placed on the concrete marina breakwater constituting a part of the building. Table 5 presents the roots of the mean square error (RMSE) for the location of checkpoints. Figure 6a is a graphical representation of the errors for individual checkpoints. The shape and colour of the respective ellipses represent the distribution of the GCP location error.

Internal camera orientation elements were taken from the database of the photogrammetric software use and optimized using autocalibration during the preliminary model development process. Detailed internal orientation element values after optimisation and standard deviation are presented in Table 6.

## 3. Results

### 3.1. Filtration of Point Clouds

**Figure 7:**
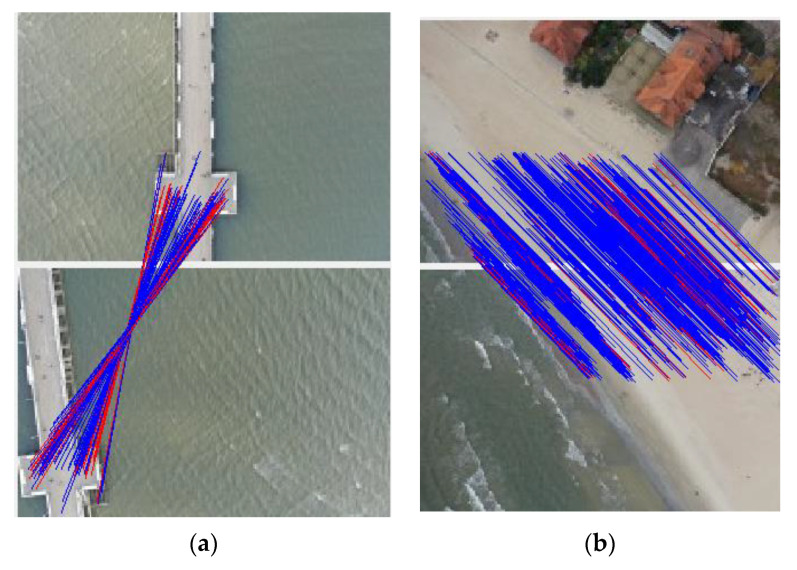
Homologous points in the water (**a**) and land (**b**) area.

It is very difficult to correctly and precisely reconstruct the water surface and it may even prove impossible in practice. This is because popular algorithms [59,60,61,62] used in photogrammetric software detection of key points in such areas are burdened with a large error. Figure 7 shows homologous points found in a stereo-pair depicting the water and land area. They clearly illustrate the problems in the reconstruction of variable surfaces. In these surfaces, the algorithm did not detect any key points. On the beach, the key points and matches are evenly distributed within the image coverage area. For clarity, Figure 7b shows images with about 800 pairs of points. In this area, an image showing 4000 pairs would be illegible. In such cases, the technique for increasing flight altitude (here used in 2018) allows obtaining a slight improvement in the surface area of the generated orthophotomap.

**Figure 8:**
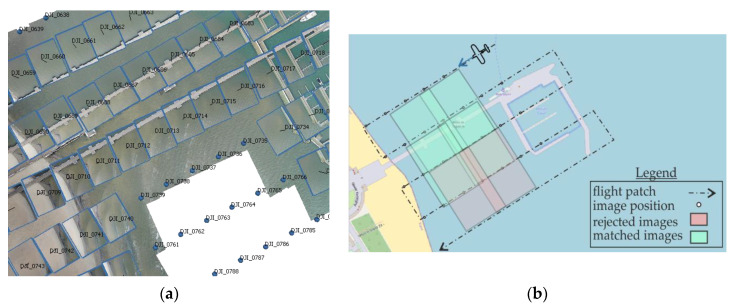
Generating an orthophotomap over the water area (**a**) during the flight in 2019, and a diagram illustrating the problem (**b**).

A rectified image may be fitted and will become part of the orthophotomap, provided that homologous points are found for a given stereo-pair. As shown in Figure 7 homologous points are not generated on the water surface. As a result, only photographs with any fixed infrastructural elements may be used for the construction of orthophotomap. Thus, around the fixed object it is only possible to generate an orthophotomap up to the maximum terrain width of the photo. The terrain size of the photo is directly proportional to the flight altitude, according to the following relationship:

(2) $$\frac{h_{MAGL}}{F_{R}}=\frac{L_{W}}{S_{W}}$$

where: $h_{MAGL}$—flight altitude (m), $F_{R}$—the actual focal length of the camera (mm), $L_{W}$—terrain width of the image and $S_{W}$—the actual width of the sensor (mm). Using this relationship, we can determine which maximum distance between the water area and fixed objects will be depicted on the orthophotomap. Figure 8a presents a fragment of the study with the aero-triangulated images and the image visible on them mapped. The described phenomenon is schematically presented in Figure 8b, where green marks the terrain area of the photo that can be included in the orthophotomap, whereas red marks the rejected photos. Fixed infrastructure elements are marked in orange.

**Figure 9:**
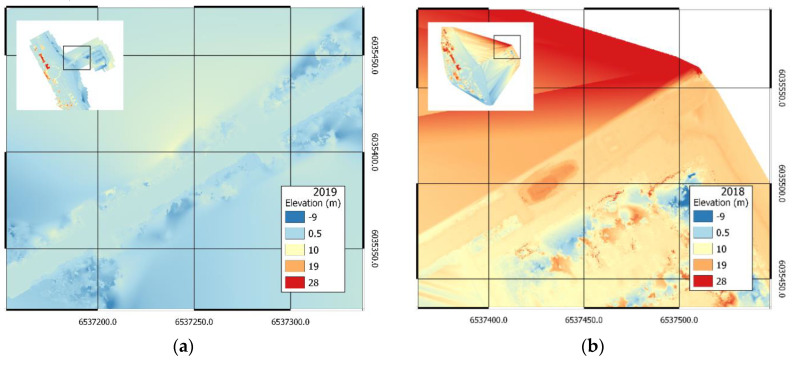
DSM - Incorrectly reconstructed altitudes (**a**) pier area 2019, (**b**) pier area, and northern area in 2018.

Incorrectly located key points affect the accuracy of the high-density point cloud; thus, other photogrammetric products have visible artefacts. In the case of DSM, the resulting ambiguities in the location of key points consist of incorrect altitude reconstruction, which is visible north of the pier in Figure 4b and near the central part of the pier itself in Figure 5b. The issue was also presented in more detail in Figure 9.

**Figure 10:**
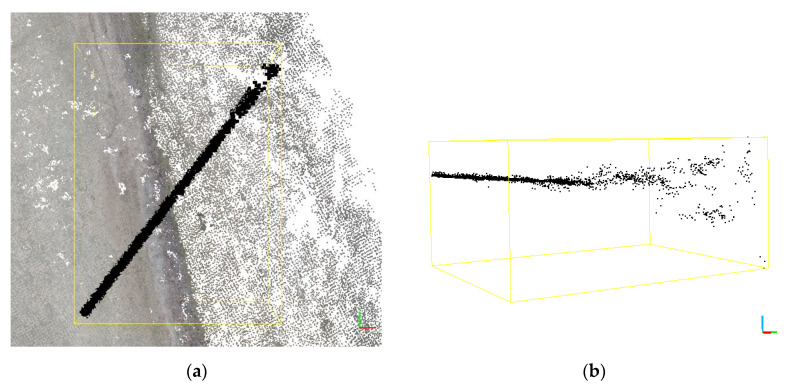
Cross-section of a high-density point cloud on the shoreline, (**a**) top view with a cross-section of the extracted cloud shown, (**b**) side view of the extracted cross-section.

If key points are incorrectly located, high-density point clouds also have a large number of additional points generated outside the mean plane determined by the reconstructed area. The number of outliers increases with a decrease in the density of key points. A fragment of the extracted cloud is shown in Figure 10. The cross-section runs on the borderline of land and water. The cross-section clearly shows the change of points distribution and their density.

This phenomenon is especially pronounced on the borderline between the luminous surface of the sea and the “dry” land. Thus, if there are several wrongly generated points located randomly outside the set of points reconstructing a given object in space, and the number of these points is significantly higher above water, then such an area can be eliminated using a statistical filter [63,64]. 

The point cloud is filtered in two phases. The first phase consists of calculating statistical data of the analysed cloud. Let us assume that each point $m_{i}$ described with coordinates $x_{i},\;y_{i},\;z_{i}$ belongs to space $R^{3}$. Thus, a point cloud before filtration M with a total number of points $M_{p}$ can be described as follows:

(3) $$M=\left\{m_{i}\right\};i=1,\ldots ,M_{p};m_{i}=(x_{i},y_{i},z_{i}).$$

Now, let’s mark the analysed point $m_{q}$ such that $m_{q}\in M$ and a point in its immediate neighbourhood $m_{n}$ such that $m_{n}\in M$. Then, a set of all *k* points in the direct neighbourhood of the point $m_{q}$ can be written as $M_{n}=\left\{m_{n1},\ldots ,m_{nk}\right\}$ on condition that each pair of points $m_{q}$ and $m_{nk}$ meets the condition:

(4) $$\sqrt[{p}]{\overset{k}{\underset{1}{\sum }}{\left|m_{nk}-m_{q}\right|}^{p}}\leq d_{m}$$

where $d_{m}$ is the maximum assumed distance between the examined point and p≥1 (here the assumed p=2).

The average distance to all k points in the neighbourhood of point $m_{q}$ is:

(5) $$d_{i}=\frac{1}{k}\overset{k}{\underset{1}{\sum }}\sqrt{{\left(m_{nk}-m_{q}\right)}^{2}}$$

and the mean value $d_{i}$ calculated for all points of the filtered cloud M is expressed by the formula:

(6) $$\mu =\sum_{i}^{M_{p}}\frac{d_{i}}{M_{p}}$$

thus, the standard deviation of distances to all k neighbourhood points for all the point $m_{i}$ can be written as:

(7) $$\sigma =\sqrt{\frac{1}{M_{p}}\;\sum_{i}^{M_{p}}{\left(d_{i}-\mu \right)}^{2}}$$

The first filtration step finishes with the calculation of statistical values. The next filtration stage consists in generating the resultant point cloud ***M****_o_*, without outliers. Outliers are points that are located at a greater distance to *k* nearest neighbours than a certain threshold value *T*. This threshold value can be defined as follows:

(8) T=μ+ασ

where *α* is a user-defined multiplier and can be determined experimentally for a given point cloud. The resulting point cloud after filtering can thus be written as follows:

(9) $$M_{o}=\left\{m_{q}\in M|\left(\mu -\alpha \sigma \right)\leq d_{i}\leq \left(\mu +\alpha \sigma \right)\right\}$$

**Figure 11:**
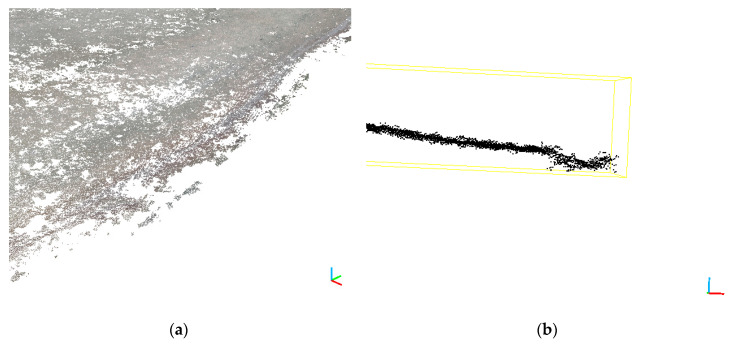
Cloud fragments cleared from water surface noise, 2018 campaign (**a**) and cross-section of the filtering point cloud (**b**).

The point clouds obtained from photogrammetric flights by iteration have been cleaned according to a procedure adopted and described above, which means that the resulting cloud from each run has been filtered again. In this way, outliers were eliminated. The number of iterations for a given set and the coefficients for each point cloud are shown in Table 7. The graphical result of the filtration described above is shown in Figure 11. Figure 11 shows the cleaned section, previously shown in Figure 10. This section clearly shows a significant reduction of outliers over the land area and a complete absence of those representing the water surface. 

### 3.2. Precision Cloud Fit

**Figure 12:**
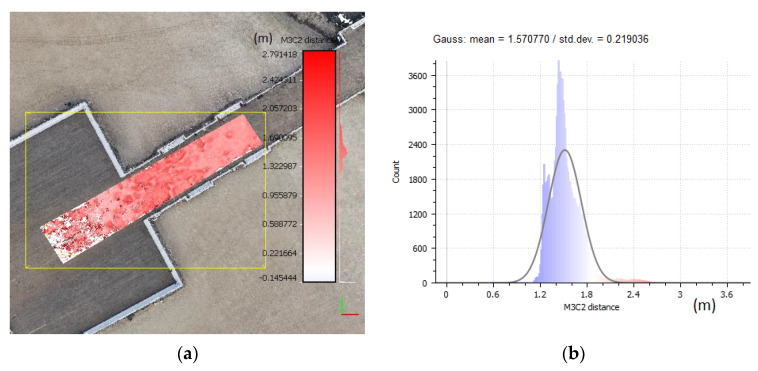
Cloud distances and noise before data filtering and fit: (**a**) the selected sample data segment, (**b**) the histogram and normal distribution (μ = 1.57 m, σ = 0.22 m).

A precise fit of the point clouds was performed on previously filtered data. The procedure for aligning the clouds was necessary since the point cloud as of 2018 was developed without GCP. The cloud created in 2019 was taken as a reference cloud. The elements of the rotation matrix and translation vector for the 2018 measurements were calculated using the ICP (iterative closest point) algorithm [65,66,67], which is commonly used for such tasks. These elements were defined in a selected data segment. The segments were selected from stable and invariant areas such as pavements along the beach and part of the promenade in front of the pier. It was assumed that such infrastructure elements did not change significantly during the analysed period. Figure 12 presents one of the object’s (pier’s) segment and the analysis of the distance of corresponding points of the clouds subject to comparison. The values correspond to the data before filtration and before the precise fit. The measurement noise present in the measured point clouds (especially visible with the 2018 data) is visible on the histogram (Figure 12b) in the form of a single peak corresponding to approximately 1.2 m. The distance differences in the test sample have a distribution close to normal with a mean of μ = 1.57 m and a standard deviation of σ = 0.21 m.

**Figure 13:**
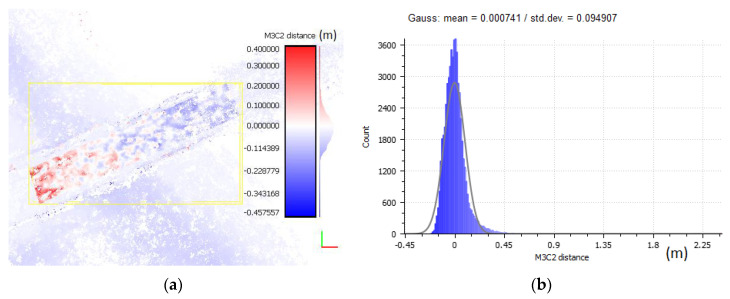
Cloud fit and noise before data filtering: (**a**) the selected data segment, (**b**) the histogram and normal distribution (μ = 0.07 mm, σ = 0.095).

The values representing the fit and filtration results are shown in Figure 13. This analysis shows that the distance differences in the test sample have a normal distribution with a mean of μ = 0.7 mm and a standard deviation of σ = 0.095 mm. This means that the mean distance difference after the fitting significantly decreased (by 1.5693 m) and the value of the mean distance difference for clouds after filtration is close to zero. The standard deviation after filtration has also decreased significantly, which means that noise (number of outliers) has been significantly reduced. The analysis of the histogram and distribution after fitting and filtration shows that no significant anomalies occur. The data is normally distributed and include a few outlier observations, and a significant part of the observations concentrates around the mean value.

### 3.3. Detection of Changes 

**Figure 14:**
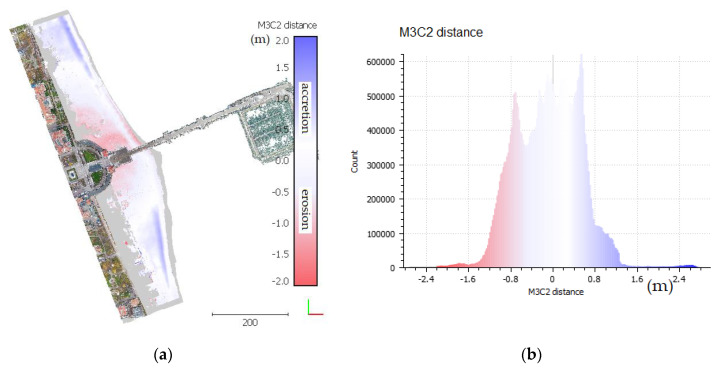
Changes in the beach area (**a**) point cloud with colour-coded change values, (**b**) distance histogram.

The M3C2 method described in [32,68] was used to determine changes occurring within the beach area. The M3C2 algorithm was designed for measure accurately 3D distances directly between two given point clouds. One of them is considered as a reference cloud, to which distance is calculated. In the preliminary step, the local normal to each point or core points are estimated on given point clouds. The core points are generally a sub-sampled version of the reference cloud. After this step, a distance along the local normal between two-point clouds is measured [68,69]. The changes that took place during the year in the beach area are illustrated in Figure 14a. The values were referred to the 2018 model and presented on the 2019 cloud points in an appropriate colour. This means that the negative values (blue) represent points of the 2019 cloud located lower on the z-axis in relation to those recorded in 2018, whereas red colour means that points from the 2019 campaign are higher on the z-axis. Figure 14b presents the empirical distribution of the observed distances between campaigns, i.e., distances between cloud points. 

Over the year, the northern and southern parts of the beach changed significantly. Sand sediment transport due to the influence of wind and undulation on the tombolo (salient) [42] just being formed causes the sand from the northern and southern regions to be transported and deposited in the beach area. The very character of the formation of coastal dunes results from close links with the coastal sand accumulation system, and the dynamics of the processes taking place there largely depend on the denudation balance of the given coastal area, the nature of coastal currents, undulations and the wind. 

The figure presented above shows new, forming of accretionary berms, up to 1 m high, formed after early autumn storms. Berm position determines the maximum range of incoming waves and its size represents the storm force. In the Baltic Sea, the berms are up to 3 m high, and on the open Atlantic coast, in places, berms are over 10 m [36,70]. 

The sand transmission caused by wind intensifies in the northern and southern parts of the studied area. Erosion dominates in the central part, near the pier entrance. Erosion in this area is caused by intensified human activity, and do not have a natural source. The direct factors include human morphological activities with the use of machines [71]. In high season, the beach is heavily exploited which forces the movement of sand from the pier neighbourhood. The reduction in the supply of material, which may be due to various reasons, changes the beach’s sedimentary budget and leads to erosion. Accelerated beach erosion is mainly due to anthropogenic pressure and results from the stabilization of parts of the beach [36], as is the case here. The stabilised platform and its surroundings limit the transport of material. At the same time, a clear change in the shoreline can be observed, especially in the central part near the pier, where the material transported by sea currents is accumulating, gradually moving towards the pier. A histogram (Figure 14b) analysis shows that the transmission of the material within the whole studied area is balanced. Erosion occurring around the recreational zone in the central part is balanced by the accumulation in the northern and southern regions.

As shown, the denudation balance of the analysed beach is constant, which means that erosion and accumulation processes counterbalance. This balance is mainly of an anthropogenic origin, and is artificially supported by human activity. Factors indirectly affecting the balance include land-based technical infrastructure and commercial buildings that promote erosion and limit material transmission in the area. Additionally, intensive tourist traffic significantly impacts the balance. This limits the natural ability of the vegetation to grow on the dunes, which intensifies sand transmission in places slightly further from the infrastructure. This artificially maintained balance requires continuous human activity.

## 4. Conclusions

This article presents the problem of low-level photogrammetry used for the measurement campaign of the unique phenomenon in Sopot. This phenomenon has been monitored for several years [72], and in the last 2 years, unmanned platforms and various spatial measurement techniques have been increasingly used for this purpose by a scientific group created from the contributors of this article. Unmanned aircraft were a part of this comprehensive measurement campaign [42] and their use was intended to gather information on changes in the beach surface. The combination of many, mutually complementary, measurement techniques allows for obtaining a broad image of the changes taking place in the littoral area. 

Various types of UAVs and other cameras were used in the two analysed cases. These platforms performed a photogrammetric flight according to different plans. Preliminary data validation showed that a direct comparison of the photogrammetric products developed over different years would result in errors and yield unreliable results. These errors are mainly due to differences in data acquisition, software used and other processing settings. Therefore, in the presented research the unification of data processing by adopting common assumptions was proposed. In this procedure, the statistical filtration was applied in order to eliminate outliers and subsequently the ICP algorithm was used to harmonise the content. This operation made possible the comparative analyses and, as a consequence, the identification of changes.

High-density point clouds, especially the one developed in 2018, exhibited a significant number of outliers. These points were eliminated by iterative application of the statistical filtration method. This reduced standard deviation, as seen in selected sections, and eliminated incorrectly reconstructed unstable surfaces. This process stage was necessary for the correct and precise mutual fit of the spatial models. In selected, stable data segments located in the studied area, the distance difference was reduced to the mean value of μ = 0.07 mm. The changes taking place on the beach surface were indicated with point clouds fitted in this way.

Natural geomorphological processes taking place in the littoral area keep changing its shape and a kind of a specific collision with the infrastructure occurs there. The wind changes the beach surface in annual cycles. This results in covering the infrastructure with sand, creating natural hollows and dunes. Tombolo (salient), which constantly changes the shoreline, also causes the water body near the marina to become shallower [44]. In the last 25 years, a constant increase in water levels due to storms has been observed in the southern Baltic Sea, which proves a continuous intensification of these phenomena [73]. Measurement techniques using unmanned platforms allow for a comprehensive assessment of such phenomena in these areas and for planning anthropopressure.

## Figures and Tables

**Table 1:** Technical data of DJI Mavic Pro and DJI Mavic Pro 2 unmanned aerial vehicles.

Technical Data	DJI Mavic PRO	DJI Mavic PRO 2
Dimensions (L × W × H) (mm)	305 × 244 × 85	322 × 242 × 84
Weight (g)	734	907
Maximum rising speed (m/s)	5	5
Maximum ascending velocity (m/s)	3	3
Maximum advance velocity (km/h)	65	72
Maximum altitude (m)	5000	6000
Maximum flight time (min)	27	31
Maximum hovering time (min)	24	29
Mean flight time (min)	21	25
Maximum flight range (km)	13	18
Permissible operating temperature range (°C)	0 to 40	−10 to 40
Satellite Navigation Systems	GPS/GLONASS	GPS/GLONASS

**Table 2:** Data of FC220 and L1D-20c (Hasselblad) cameras.

Technical Data	F230	L1D-20c (Hasselblad)
Sensor size	1/2.3”, 12.35 MP	1”, 20 MP
Pixel size (μm)	1.55	2.41
Lenses (Field of vision—FOV)	FOV 78.8° (28 mm ^1^) f/2.2	FOV 77° (28 mm ^1^) f/2.2
Focus	from 0.5 m to ∞, auto/manual focus	from 1 m to ∞, auto/manual focus
ISO sensitivity range	100–3200 (video), 100–1600 (photo)	100–6400 (video), 100–12,800 (photo)
Electronic shutter time	8 s–1/8000 s	8 s–1/8000 s
Image size (pixel)	4000 × 3000	5472 × 3648
Photo modes	Single shot, Burst shooting: 3/5/7 frames, Auto Exposure Bracketing (AEB): 3/5 bracketed frames at 0.7 EV, Interval	Single shot, Burst shooting: 3/5 frames, Auto Exposure Bracketing (AEB): 3/5 bracketed frames at 0.7 EV, Interval
Video modes	C4K: 4096 × 2160, 24 fps	4K: 3840 × 2160 24/25/30 p
	4K: 3840 × 2160, 24/25/30 fps	2.7K: 2688 × 1512
	2.7K: 2720 × 1530, 24/25/30 fps	24/25/30/48/50/60 p
	FHD: 1920 × 1080, 24/25/30/48/50/60/96 fps	FHD: 1920 × 1080
	HD: 1280 × 720, 24/25/30/48/50/60/120 fps	24/25/30/48/50/60/120 p
Image file format		JPEG, DNG
Video file format	MP4, MOV (MPEG-4 AVC/H.264)	MP4/MOV (MPEG-4 AVC/H.264, HEVC/H.265)

^1^*35 mm format equivalent*.

**Table 3:** Flight details.

	2018 Priority Area	2018 Auxiliary Area	2019
Flight path	Double grid	Free flight	Single grid
Ground sampling distance (GSD)	2.25	8.4	2.21
Number of photos taken	621	413	462
Coverage (longitudinal/traverse) (%)	80/80	85–95/85–95	65/65
Flight altitude above ground level (AGL)	60	150–260	100

**Table 4:** Data obtained from reports generated by photogrammetric software.

Parameter	2018	2019
Ground control points (GCP)	No	Yes
Total number of images with georeferencing	1037	462
Number of images used for modelling	964	233
GCP measurement accuracy	NA *	RTK GPS
Mean reprojection error (pix)	0.301	0.514
Total number of TPs connection points (3D)	1,454,125	218,226
Median of key points per image	19,475	40,000
Direct georeference	GPS	GPS
Median matches per image	4717.91	4000
Mean absolute camera position error (x,y,x) (m)	0.165, 0.167, 0.272	0.597, 2.205, 65.441
Mean camera position standard deviation (x,y,z) σ	0.052, 0.059, 0.067	0.020, 0.018, 0.014
Number of points in dense point cloud	21,765,551	116,831,423

* DG: double grid, SG: single grid, Free: manually operated flight, NA: not available.

**Table 5:** Mean square errors in the location of ground control points.

Number of GCP	RMSE X (cm)	RMSE Y (cm)	RMSE Z (cm)	RMSE XY (cm)	Total RMSE (cm)
7	7.02742	4.46724	0.705093	8.32712	8.35692

**Table 6:** Internal camera orientation elements.

Camera	F (pix)	C_x_ (pix)	C_y_ (pix)	K_1_	K_2_	K_3_	P_1_	P_2_
F220	2808.897	1956.756	1498.347	0.040	−0.128	0.118	0.000	0.000
σ	0.415	0.089	0.082	0	0	0	0	0
L10	4256	2691.02	1803.22	−0.0182	−0.0168	0.0125	−0.00236	−0.00139
σ	0.401	0.13	0.063	0.000034	0.00013	0.00015	0.0000024	0.0000023

**Table 7:** Number of iterations and adopted coefficients.

Campaign	Coefficient	1	2	3	4	5	6
2018	*k*	6	6	6	6		
	α	1	1	1	1		
2019	*k*	6	6	6	6	6	6
	α	1	1	1	1	1	1
